# RNA folding on the 3D triangular lattice

**DOI:** 10.1186/1471-2105-10-369

**Published:** 2009-11-05

**Authors:** Joel Gillespie, Martin Mayne, Minghui Jiang

**Affiliations:** 1 Department of Computer Science, Utah State University, Logan, Utah 84322-4205, USA

## Abstract

**Background:**

Difficult problems in structural bioinformatics are often studied in simple exact models to gain insights and to derive general principles. Protein folding, for example, has long been studied in the lattice model. Recently, researchers have also begun to apply the lattice model to the study of RNA folding.

**Results:**

We present a novel method for predicting RNA secondary structures with pseudoknots: first simulate the folding dynamics of the RNA sequence on the 3D triangular lattice, next extract and select a set of disjoint base pairs from the best lattice conformation found by the folding simulation. Experiments on sequences from PseudoBase show that our prediction method outperforms the HotKnot algorithm of Ren, Rastegari, Condon and Hoos, a leading method for RNA pseudoknot prediction. Our method for RNA secondary structure prediction can be adapted into an efficient reconstruction method that, given an RNA sequence and an associated secondary structure, finds a conformation of the sequence on the 3D triangular lattice that realizes the base pairs in the secondary structure. We implemented a suite of computer programs for the simulation and visualization of RNA folding on the 3D triangular lattice. These programs come with detailed documentation and are accessible from the companion website of this paper at http://www.cs.usu.edu/~mjiang/rna/DeltaIS/.

**Conclusion:**

Folding simulation on the 3D triangular lattice is effective method for RNA secondary structure prediction and lattice conformation reconstruction. The visualization software for the lattice conformations of RNA structures is a valuable tool for the study of RNA folding and is a great pedagogic device.

## Background

Difficult problems in structural bioinformatics are often studied in simple exact models to gain insights and to derive general principles. Protein folding, for example, has long been studied in the lattice model [1-4]. Recently, researchers have also begun to apply the lattice model to the study of RNA folding [5-10]. In the lattice model, the folding of a biopolymer takes place on a lattice: each monomer occupies a unique lattice point; consecutive monomers in the sequence occupy adjacent lattice points. The structure prediction problem then reduces to the simplified problem of finding a lattice conformation of the biopolymer to achieve certain desirable properties: for protein folding, to maximize the number of contacts between hydrophobic amino acids; for RNA folding, to maximize the number of hydrogen bonds between complementary bases. The simplified problem does not reflect the full reality of underlying biological process, but at least gives a first-order approximation and is more tractable. Schuster [11] remarked that "RNA secondary structures, together with lattice protein models, are at present the only biological objects for which conformational landscapes and sequence-structure maps can be computed and analyzed in sufficient detail."

In this paper, we present a method for predicting RNA secondary structures with pseudoknots by folding simulation on the 3D triangular lattice. A drawback prevalent in the existing models for RNA secondary structure prediction is that they are static: the predicted secondary structures are selected only because they have the lowest free energies. But RNA folding is a dynamic process. RNA pseudoknots that are too complex are difficult to realize naturally because of the barriers in the energy landscapes due to the steric constraints of excluded volume. To cope with this problem, various restrictions may be imposed on the predicted secondary structures; for example, the hairpin constraint requires two bases forming a base pair be separated by at least three other bases in the sequence. The steric constraints of excluded volume, however, are inherently geometric; it is impossible to capture them all with only a few combinatorial restrictions. Our method overcomes this difficulty by simulating the folding of RNA tertiary structures on the 3D triangular lattice directly, as a dynamic process; the secondary structures are then extracted from the tertiary structures. Folding simulation can be computationally intensive (that is, quite slow) because of its exaustive-search nature, especially for complex models that include considerable details. Thus the simulation of RNA folding dynamics has only been used in mapping and analyzing the energy landscapes of certain small RNAs [12,13]. Nevertheless we show that by using a simple lattice model and an efficient move set, and by careful algorithmic engineering, folding simulation can be turned into an effective method for RNA secondary structure prediction. Experiments on sequences from PseudoBase [14] show that for short RNA sequences, our method is even more accurate than the HotKnot algorithm of Ren, Rastegari, Condon and Hoos [15], a leading method for predicting RNA secondary structures with pseudoknots. In the context of protein folding, the recovery of protein tertiary structures from contact maps has been studied by many researchers [16-20]. The analogous problem of reconstructing RNA tertiary structures from secondary structures, however, has not yet been seriously considered. This is understandable because a polynucleotide chain is not as rigid as a polypeptide chain: the RNA secondary structure alone does not contain enough spatial information to decide the RNA tertiary structure. Nevertheless, a method for reconstructing a 3D conformation of an RNA sequence from a given list of base pairs is still valuable, at least for validating predicted secondary structures, considering that some secondary structures are so unnatural that they cannot be completely realized in 3D due to the steric constraints. We will show that our method for RNA secondary structure prediction can be adapted to a very efficient method for RNA tertiary structure reconstruction in this limited sense.

RNA secondary structures can be visually represented in many different ways. Besides the simple representations of the dot-bracket notation, the Feynman diagram, and the dot plot, most RNA software packages such as Mfold [21,22] and Vienna [23,24] include programs that visualize the RNA classical structures [25] using flat drawings in the plane. Advanced visualization tools such as RnaViz [26], PseudoViewer [27], and jViz.Rna [28,29] can even generate flat drawings of RNA secondary structures with pseudoknots. In such flat drawings, each RNA base is represented by a letter, consecutive bases in the sequence and base pairs in the secondary structure are then connected by edges between the letters. Ideally, the bases should be placed such that the edges connecting them are straight and of uniform length, to signify their geometric proximity. Also, crossing edges are undesirable and should be avoided. But real RNA structures are not flat. For example, the pseudoknot of regulatory region of alpha ribosomal protein operon of E. coli (PKB00071 from PseudoBase [14]) has the following secondary structure (in dot-bracket notation) that includes three groups of inter-crossing base pairs:

UGUGCGUUUCCAUUUGAGUAUCCUGAA

(((((((:(((((::::::::[[[[::

AACGGGCUUUUCAGCAUGGAACGUACA

::[[[[::::{{{{:))))))))))))

UAUUAAAUAGUAGGAGUGCAUAGUGGC

::::::::::::::::::::::::::]

CCGUAUAGCAGGCAUUAACAUUCCUGA

]]]:::::]]]]:::::::::::}}}}

Despite the best effort, flat drawings of such complicated secondary structures will inevitably contain crossing edges or edges of different lengths that distort the true geometric structures. The simulation of RNA folding on the 3D triangular lattice provides an alternative method for the visualization of RNA secondary structures. Given a secondary structure of an RNA sequence, we can first reconstruct a lattice conformation of the sequence that realizes the base pairs in the secondary structure, then display the lattice conformation using a 3D visualization software. The lattice conformation of an RNA secondary structure may not represent the true tertiary structure, but at least it preserves geometric properties such as proportion and distance and hence looks more realistic.

We implemented a suite of computer programs for the simulation and visualization of RNA folding on the 3D triangular lattice. These programs come with detailed documentation and are accessible from the companion website of this paper: **RNA folding on the 3D triangular lattice **http://www.cs.usu.edu/~mjiang/rna/DeltaIS/.

## The 3D Triangular Lattice

In this section, we introduce the 3D triangular lattice and explain our choice of this particular lattice over other alternatives.

**Figure 1:**
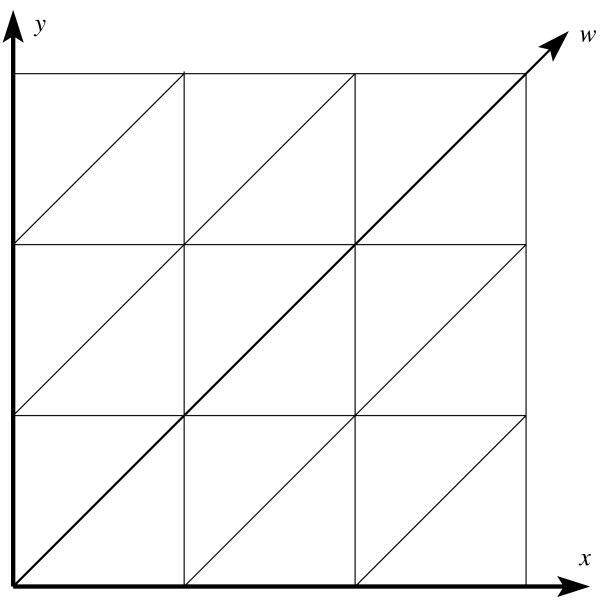
**Adding an auxiliary axis along the diagonal of a square lattice**.

**Figure 2:**
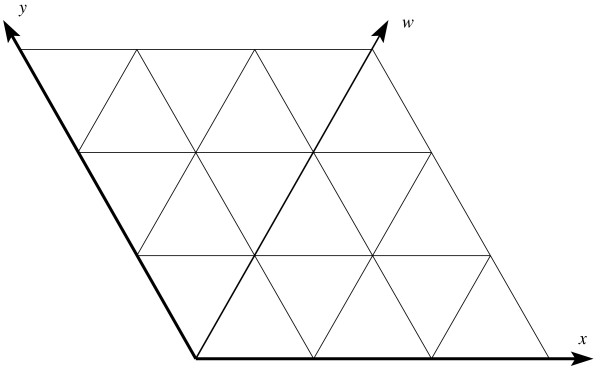
**Skewing the square lattice into a 2D triangular lattice**.

We first examine the 2D triangular lattice as a warm-up exercise. Refer to Figures 1 and 2. Let  and  be the two primary axes of the square lattice. Add an auxiliary axis  along the *xy *diagonal, then skew the square lattice until the angle between  and  becomes 120°, and we obtain the 2D triangular lattice. On the 2D triangular lattice, each lattice point can be specified by its coordinates along the two primary axes  and . For example, the lattice point  = (*x, y*) has six neighbors (*x *+ 1, *y*), (*x *- 1, *y*), (*x*, *y *+ 1), (*x, y *- 1), (*x *+ 1, *y *+ 1), and (*x *- 1, *y *- 1). Using the auxiliary axis  besides the two primary axes  and , the six neighbors of  can be more succinctly represented as , , and . We now examine the 3D triangular lattice. There are two common types of 3D triangular lattices: the CCP lattice, formed by a cubic closest packing of spheres, consists of layers of 2D triangular lattices repeating in an ABCABC pattern; the HCP lattice, formed by a hexagonal closest packing of spheres, consists of layers of 2D triangular lattices repeating in an ABAB pattern. We use the CCP lattice.

**Figure 3:**
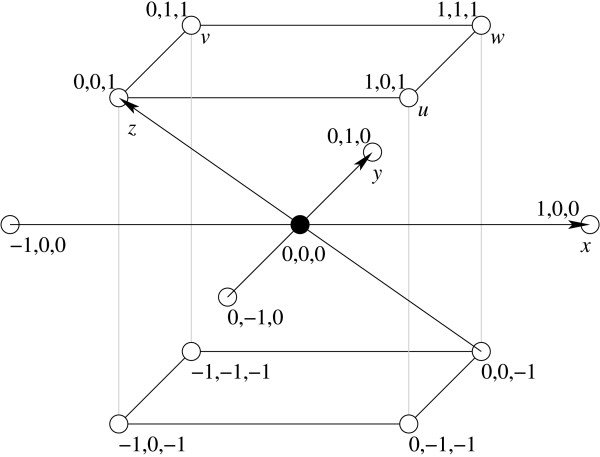
**The 3D triangular lattice**. Each lattice point has 12 neighbors distributed among three consecutive layers of square lattices in parallel planes, four neighbors on each layer.

**Figure 4:**
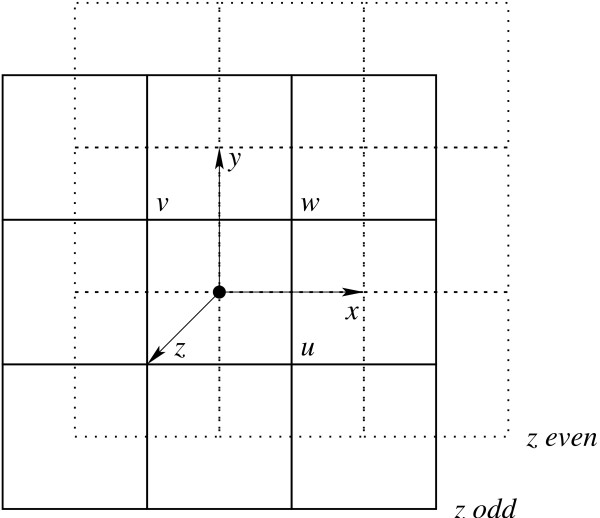
**A projected view of the 3D triangular lattice**. Alternating layers of square lattices are depicted by solid and dotted lines.

Refer to Figure 3. Besides the two primary axes  and  of the square lattice, the 3D triangular lattice has an additional primary axis  pointing outside the *xy *plane at an angle of 120° from both  and . Refer also to Figure 4. An interesting property of the (CCP) 3D triangular lattice is that, from one perspective, it can be viewed as layers of 2D triangular lattices repeating in an ABCABC pattern, but from another perspective, it can be viewed as layers of square lattices repeating in an ABAB pattern. The distance between two parallel planes supporting two consecutive layers of square lattices is exactly  units of the square lattice. On the 3D triangular lattice, each lattice point can be specified by its coordinates along the three primary axes , , and . For convenience, we also define three auxiliary axes , , and . Then each lattice point  has exactly 12 neighbors , , , , , and . Using the three auxiliary axes besides the three primary axes, the lattice conformation of an RNA sequence *S *of *n *bases can be represented as a *turn sequence T *of *n *- 1 directions over the alphabet  that simulates a self-avoiding walk on the lattice.

Many types of lattices have been proposed by researchers in structural bioinformatics. Besides the 2D and 3D triangular lattices [30,10] and the very common square and cubic lattices [1,31,32], we also have the 2D hexagonal lattice [33], the diamond lattice [34-37], the face-centered cubic lattice [38], and the (2, 1, 0) lattice [39-41]. Our choice of the 3D triangular lattice for RNA folding is based on three considerations:

**Regularity**: On the 3D triangular lattice, all lattice points have the same number of neighbors, and all pairs of adjacent lattice points have the same distance. This allows a uniform handling of geometric proximity in base pairing.

**Density**: The *coordination number *of a lattice is the number of neighbors of each lattice point. The 3D triangular lattice has a coordination number of 12; the cubic lattice has a coordination number of only 6. Thus the 3D triangular lattice is better than the cubic lattice in approximating the 3D space. Indeed the 3D triangular lattice is the densest regular lattice in 3D. There exist, admittedly, alternative 3D lattices with even higher coordination numbers. The (2, 1, 0) lattice (which models chess moves of a knight in 3D) and other extensions of the cubic lattice by Skolnick et al. [39-41] are notable examples. Such lattices are irregular however. At the expense of regularity, the 3D triangular lattice can be similarly extended to acquire higher coordination numbers.

**Parity**: Each element of a sequence has a *parity *of either odd or even depending on its position in the sequence. When an RNA sequence folds on the cubic lattice, complementary bases of the same parity can never become adjacent to form base pairs. This unnatural constraint is intrinsic to the cubic lattice and renders it unsuitable for folding simulation. The 3D triangular lattice is not susceptible to such parity problems.

These considerations has led us to believe that for folding simulation the 3D triangular lattice is superior to the other 3D lattices. Indeed the cubic lattice and its variants are popular mainly because of the ease of programming on such lattices. Our previous mathematical exposition shows that, with the auxiliary axes, the 3D triangular lattice is not much more difficult to implement than the cubic lattice. We hope that our first use of the 3D triangular lattice will encourage its wider adoption by the other researchers. For example, the recent work by Cao and Chen on predicting RNA pseudoknot folding thermodynamics [37] could benefit from a switch from the (irregular) diamond lattice to the 3D triangular lattice.

## Methods

In this section, we present our method for RNA folding on the 3D triangular lattice. The following discussion focuses on a novel method for predicting RNA secondary structures with pseudoknots, which works in two stages: first simulate the folding dynamics of the RNA sequence on the 3D triangular lattice, next extract and select a set of base pairs from the best lattice conformation found by the folding simulation. We then adapt this method for RNA secondary structure prediction to a method for RNA tertiary structure reconstruction, and introduce our 3D visualization program for RNA folding.

### Simulating RNA Folding Dynamics on the 3D Triangular Lattice

We use the classical combinatorial optimization technique of simulated annealing [42,43] for the simulation of RNA folding dynamics on the 3D triangular lattice. In the following, we first introduce the basic annealing procedure, next describe the move set for transforming the lattice conformations and the scoring function for each lattice conformation, then discuss three techniques that improve the efficiency and effectiveness of the folding simulation.

#### Basic Annealing Procedure

A basic iteration of the annealing procedure goes as follows. In the beginning, the sequence is initialized to an arbitrary lattice conformation *v*_0_. Then, in each step *i*, a candidate move is selected at random to transform the old conformation *v*_*i*-1 _into a new conformation *v*_*i*_. Each conformation *v *has a corresponding score *s*(*v*) ≥ 0. If *s*(*v*_*i*_) ≥ *s*(*v*_*i*-1_), then the move is accepted and the annealing procedure continues to the next step. Otherwise, the move is either accepted with a probability of *p*_*i *_or rejected with a probability of 1 - *p*_*i*_. The acceptance probability  depends on both the score difference *s*(*v*_*i*_) - *s*(*v*_*i*-1_) and the system temperature *T*_*i*_. Given a number *n *of annealing steps, a cooling schedule of *T*_*i *_= *c*/log_2_(1 + *i*/*n*) is adopted because of its desirable statistical properties [44]. When *i *= 0, *T*_0 _= ∞; when *i *= *n*, *T*_*n *_= *c*. Thus the system slowly cools down as the annealing procedure progresses from step 1 to step *n*. The constant *c *is set to 1/log_2 _10 so that, as the system temperature *T*_*i *_drops from ∞ to *c*, the acceptance probability *p*_*i *_drops correspondingly from 1 to about  for a score difference of -1. In particular, since all random moves are accepted with probability close to 1 during the initial stage of the annealing procedure, the starting conformation is quickly tranformed into some random configuration (thus the choice of any special starting conformation is irrelevant). The physical interpretation of the annealing procedure is that the system starts in any conformation with equal probability at high temperature, and tends toward a good conformation at low temperature.

Note that the acceptance probability  in our annealing procedure is adapted from the probability  in the classical Metropolis criterion. The sign in the exponent is reversed because we use positive scores instead of negative energies. For convenience, we use 2 instead of *e *as the base of the exponent. Since  the coefficient of log_2_*e *is absorbed in the tunable constant *c *in the definition of *T*_*i*_.

#### Move Set

**Figure 5:**
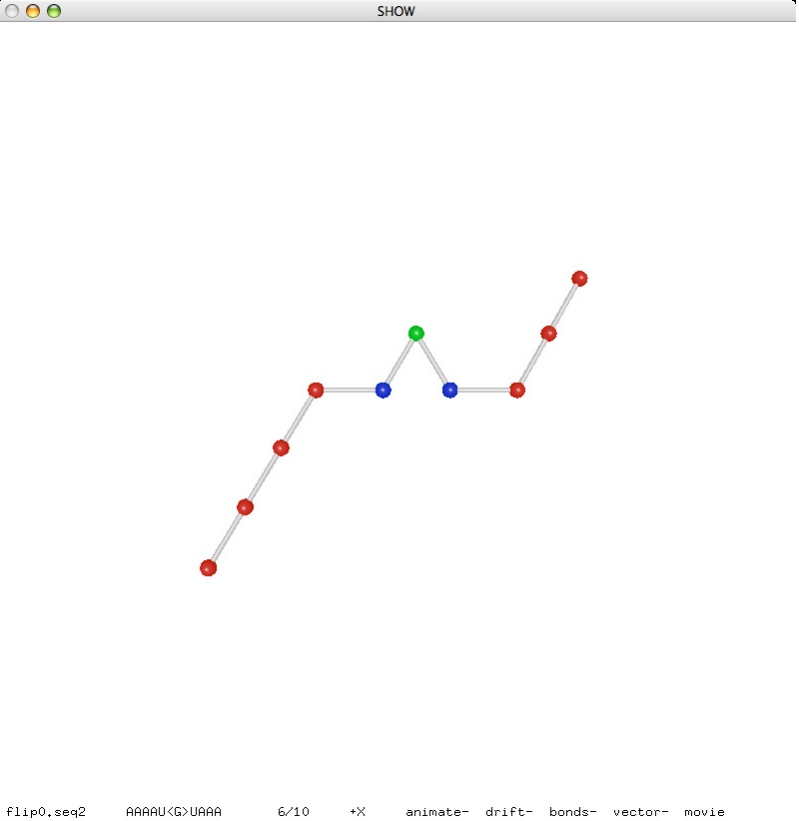
**A flip move (before)**.

**Figure 6:**
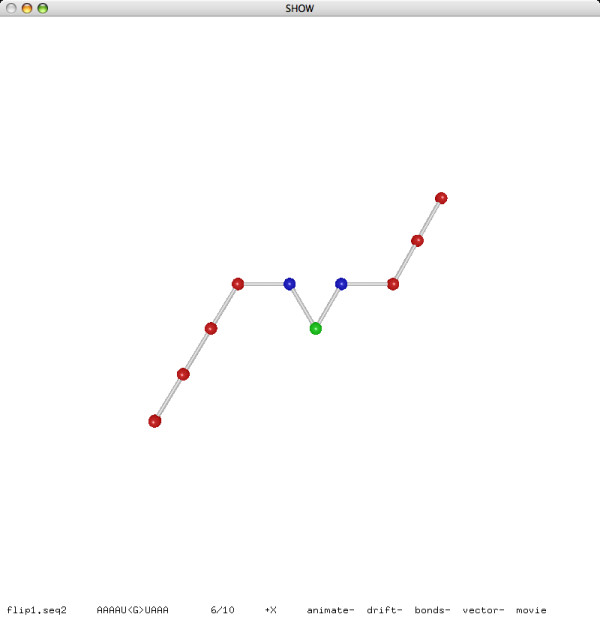
**A flip move (after)**.

**Figure 7:**
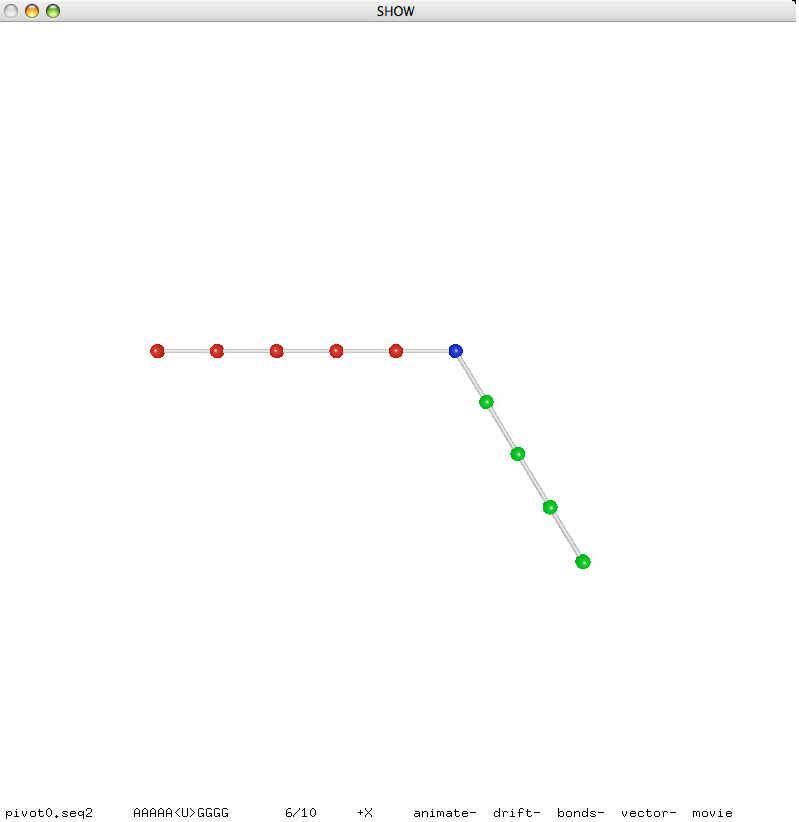
**A pivot move (before)**.

**Figure 8:**
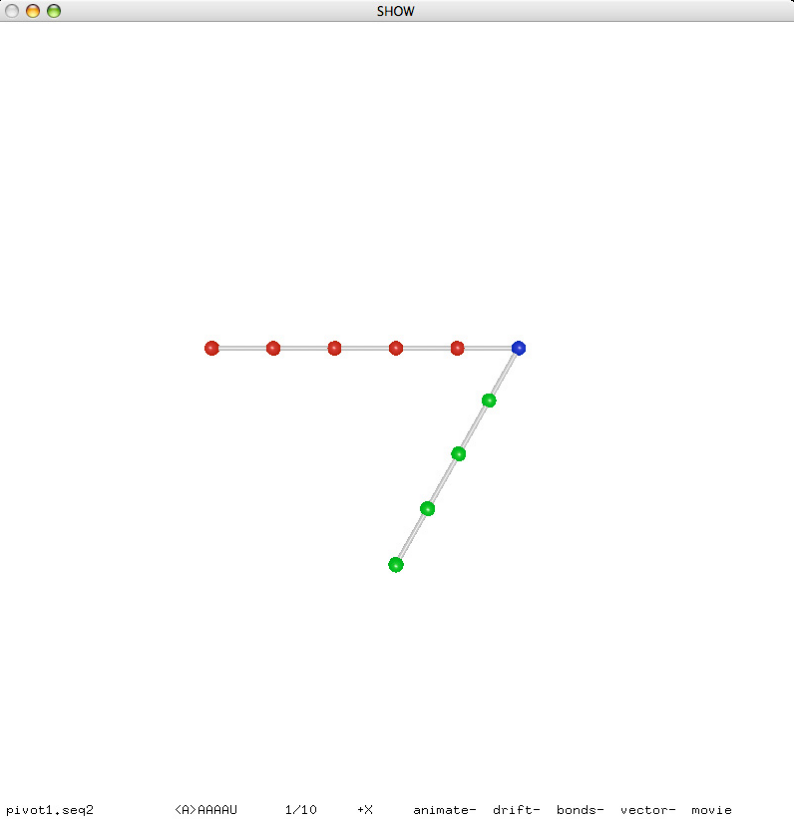
**A pivot move (after)**.

To simulate the folding of biopolymers on a lattice, we need to define a move set for structural manipulation. Two types of moves are often used for self-avoiding walks on lattices: flip moves (Figures 5 and 6) and pivot moves (Figures 7 and 8). Flip moves are local and typically operate on a small number of consecutive elements in the sequence (for example, on the square lattice, a corner move relocates a single element, and a crankshaft move relocates two consecutive elements [45]). Pivot moves [46-48], on the other hand, are global and can affect on average half of the sequence, thus incurring a high running time in folding simulation. We note that Kennedy [48] has invented a faster implementation of pivot moves that runs in *O*(*L*^*α*^) time per move on the cubic lattice, where *α *≈ 0.9 and *L *is the sequence length. But even this sub-linear running time is still too high, especially considering the large number of moves necessary for folding simulation.

**Figure 9:**
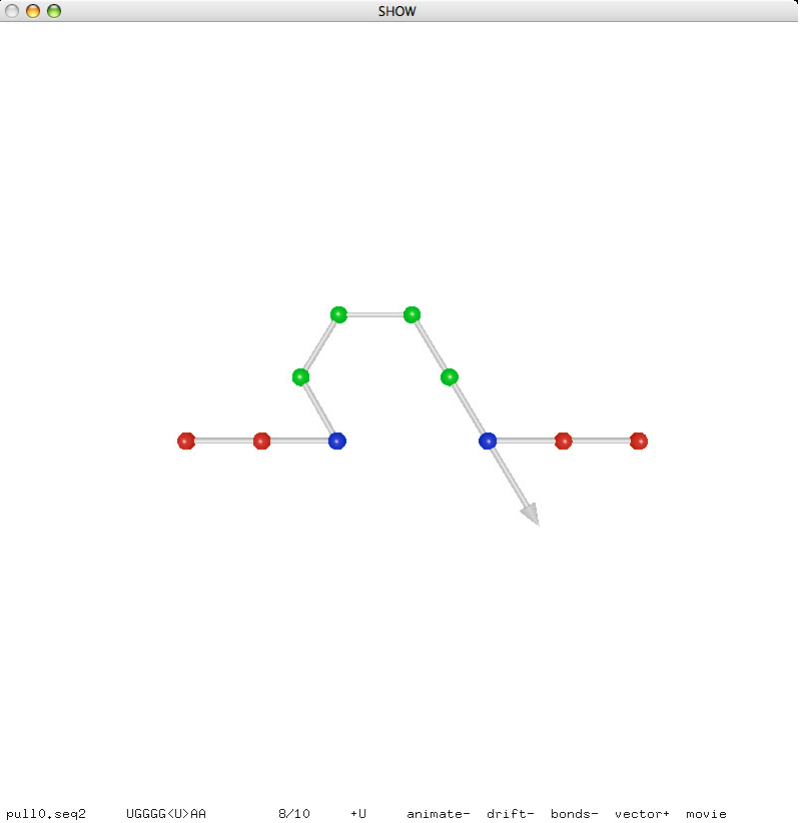
**A pull move (before)**.

**Figure 10:**
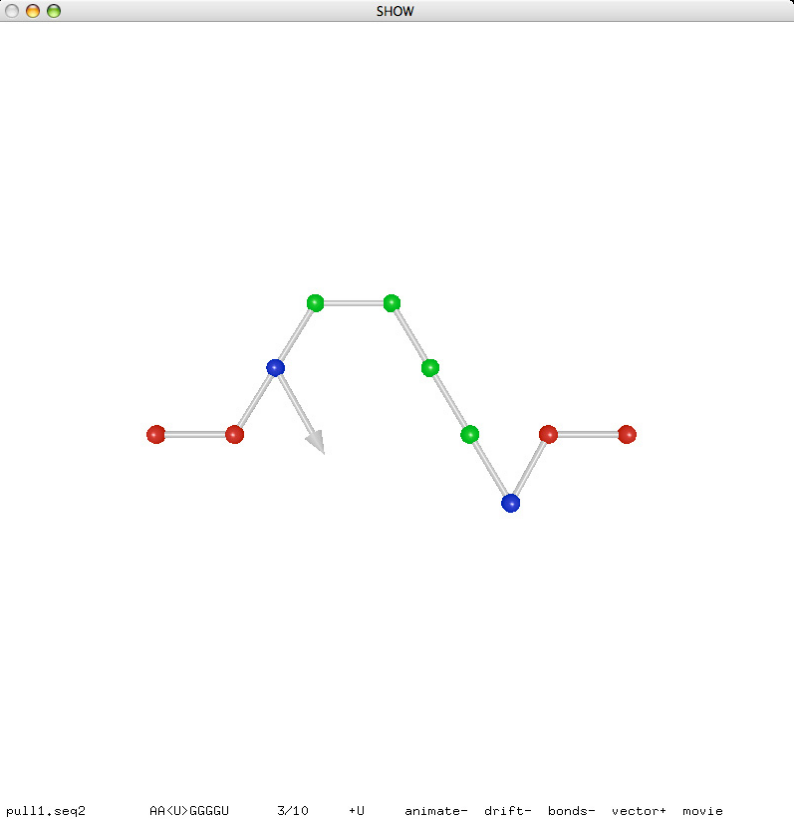
**A pull move (after)**.

We use pull moves for structural manipulation. Pull moves were used by Lesh, Mitzenmacher and Whitesides [49] for protein folding on the square lattice, and were later adapted by Jiang and Zhu [33] to the hexagonal lattice. We here adapt pull moves to the 3D triangular lattice. Refer to Figures 9 and 10 for an example. As we pull an element *e*_1 _to an adjacent lattice point, a gap may appear between *e*_1 _and a consecutive element *e*_2_. To fill this gap, we may shift *e*_2 _to the former location of *e*_1_, but then a new gap may appear between *e*_2 _and, say, *e*_3_. Repeat the shift step until an element *e*_*k*_, after being shifted to the former location of *e*_*k*-1_, remains adjacent to *e*_*k*+1 _and no more gaps appear. This whole sequence of movements is called a pull move.

Pull moves are *reversible*: each pull move that transforms a conformation *A *to another conformation *B *has a corresponding pull move that transforms *B *back to *A*. Pull moves are also *complete*: any conformation can be transformed to any other conformation by a sequence of pull moves. The complete property essentially follows from the reversible property: observe that any conformation can be easily transformed into a simple conformation in which all elements are in a straight line.

Pull moves are clearly more powerful than flip moves: on the 3D triangular lattice, flip moves are only a special case of pull moves. Moreover, pull moves have a *semi-local *property that makes them very efficient in folding simulation: although each pull move may relocate a large number of elements in the worst case (as a pivot move), the average number of relocated elements is only a small constant (as a flip move). This semi-local property, first observed by Lesh, Mitzenmacher and Whitesides [49] for pull moves on the square lattice, was confirmed by our experiments [10] for pull moves on the 3D triangular lattice. Refer to Table 1. For various sequence lengths ranging from 32 to 512, which are typical of biopolymers such as RNAs and proteins, the average numbers of elements relocated by random pull moves on the 3D triangular lattice are small constants.

**Table 1:** Numbers of relocated elements (average ± standard deviation) for various sequence lengths.

Sequence length	Number of relocated elements
32	2.4 ± 1.3
64	3.8 ± 2.7
128	4.1 ± 3.2
256	3.4 ± 2.0
512	3.5 ± 2.4

Another important feature of pull moves is their biological relevance. In certain aspects, pull moves are very similar to the shift moves proposed by Flamm, Fontana, Hofacker and Schuster [12] in their study of RNA folding kinetics by direct manipulation of base pairings (note however that shift moves are defined only for secondary structures consisting of non-crossing base pairs, while pull moves can generate secondary structures with pseudoknots). Flamm, Fontana, Hofacker and Schuster showed that shift moves (by the same token, pull moves too) allow a fast chain sliding process that closely models an important mechanism in RNA folding dynamics called defect diffusion.

#### Scoring Function

According to the Tinoco model [50], an RNA structure can be recursively decomposed into loops with independent free energy. The free energy of each loop is an affne function in the number of unpaired bases and the number of interior base pairs. Stacking pairs are the only type of loops without unpaired bases; they have negative free energy and stabilize the RNA structure. Our scoring function is designed to approximate the magnitude of total free energy contributed by the stacking pairs in a lattice conformation. This energy model is not the most accurate to date, but it captures the predominant factor and allows a fast implementation.

On the 3D triangular lattice, each lattice point has 12 neighbors. Thus in certain lattice conformations some bases may be adjacent to two or more complementary bases, although biologically each base of an RNA sequence can participate in at most one base pair. To avoid the expensive computation of a maximum-weight matching [51] for the actual base pairings in a lattice conformation, our scoring function simply computes a sum Σ_*i *_score (*i*) over all bases *i *in the sequence, where each score(*i*) is computed by examining the local neighborhood of the base *i *on the lattice. More precisely, we set score(*i*) = min_*j *_score(*i*, *j*), where *j *ranges over all complementary bases adjacent to *i*, and each score(*i*,*j*) depends on possible stackings of the three pairs (*i *- 1, *j *+ 1), (*i*, *j*), and (*i *+ 1, *j *- 1):

1. If (*i*, *j*) and (*i *- 1, *j *+ 1) are both Watson-Crick pairs, then score(*i, j*) includes the absolute value of their stacking energy (using the nearest-neighbor free energy parameters from Mfold [21,22]). Similarly, If (*i, j*) and (*i + 1*, *j - 1*) are both Watson-Crick pairs, then score(*i, j*) includes the absolute value of their stacking energy.

2. If (*i, j*) is a wobble pair and the two pairs (*i *- 1, *j *+ 1) and (*i *+ 1, *j *- 1) are both Watson-Crick pairs, then score(*i, j*) includes the doubled absolute value of the stacking energy of (*i, j*) and (*i *- 1, *j *+ 1) and the stacking energy of (*i, j*) and (*i *+ 1, *j *- 1).

The unequal treatments of wobble pairs and Watson-Crick pairs here are motivated by the biological fact that wobble pairs are rather weak and thus have little contribution to the stability of a secondary structure unless stacked with the stronger Watson-Crick pairs. In the calculation of score(*i*), we deliberately use min_*j *_score(*i*, *j*) instead of max_*j *_score(*i*, *j*) or Σ_*j *_score(*i*, *j*) to discourage clusters of base pairings that are unrealistically tight.

By using the stacking energy of consecutive base pairs, the scoring function implicitly encourages longer helices (stems). For example, the four consecutive base pairs (*i, j*), (*i *+ 1, *j *- 1), (*i *+ 2, *j *- 2), (*i *+ 3, *j *- 3) in a long helix of length four have three stackings, while the same number of base pairs (*i, j*), (*i *+ 1, *j *- 1), (*i *+ 3, *j *- 3), (*i *+ 4, *j *- 4) in two shorter helices of length two have only two stackings.

#### Optimizing Annealing Steps

The lattice conformation of an RNA sequence of *n *bases can be represented compactly as a turn sequence of *n *- 1 directions along the lattice axes. This representation is ideal for storing lattice conformations externally as files, but is not suitable for computing the scores of lattice conformations, a repeated task in simulated annealing. In our implementation, the lattice conformation is stored as an array of lattice coordinates organized into a hashtable, which is updated after every pull move. Using the hashtable, collision detection and neighborhood exploration can be performed very efficiently. Recall that the average number of bases relocated by a random pull move is a small constant. Also observe that the score difference of a conformation change due to a pull move depends on only the relocated bases and their neighbors. Thus we optimize the computation of the score difference so that each annealing step takes on average only constant time. Without this optimization, the running time of each annealing step would be linear in the length of the RNA sequence, and the folding simulation would take much longer time on longer sequences.

#### Mixing Two Cooling Schedules

The basic cooling schedule of our annealing procedure is a slowly decreasing function *T*_*i *_= *c*/*log*_2_(1 + *i*/*n*), where *n *is the total number of annealing steps. Schmitz and Steger [43] observed that certain annealing procedures can be improved by sampling the system temperatures from the basic cooling schedule at random, instead of following the decreasing function faithfully from start to finish. More precisely, in each annealing step *i*, the randomized cooling schedule chooses a random number *i' *between 1 and *n*, then uses *T*_*i' *_instead of *T*_*i *_in the calculation of the acceptance probability.

In our experimentation with both cooling schedules, we indeed observed that, given the same running time, the randomized cooling schedule is slightly more effective than the basic cooling schedule. Moreover, we observed that even better performance can be achieved by mixing the two cooling schedules together. We eventually settled on a *mixed *cooling schedule that, in each annealing step *i*, chooses either *T*_*i *_or *T*_*i' *_at random, with equal probability, as the system temperature.

#### Multiple Iterations with Doubling Steps

Because of the random nature of the simulated annealing approach, the best conformation found by the annealing procedure may vary from one iteration to another. To predict the secondary structure of an RNA sequence reliably, we repeat five iterations of the annealing procedure, then take the best among the best conformations of the five iterations. For an RNA sequence of length *L*, the number of annealing steps in each iteration is set to 100·*L*^2 ^initially. This number may be sufficient for some sequences but not for others, depending the complexity of the underlying folding landscape. We use a doubling strategy to estimate the ideal number of annealing steps. After the initial round of five iterations, we execute a second round of five iterations with the number of annealing steps doubled. This doubling process continues round after round until the improvement (in the best score) of the current round over the previous round drops below 2%. Note that 1 + 2 + 4 + ⋯ + 2^*k *^= 2^*k*+1 ^- 1 < 2·2^*k*^. Thus the total running time of the multiple rounds in this doubling process is at most two times the running time of the last round. We are not aware of any previous usage of this doubling technique in bioinformatics. But in theoretical computer science, this technique is folklore. For example, this technique has been used to estimate the number of points on the convex hull in Chan's output-sensitive convex hull algorithm [52].

### Selecting Disjoint Base Pairs for Secondary Structure Prediction

Given the best lattice conformation of an RNA sequence found by repeated annealing procedures, we need to determine a set of base pairs as the predicted secondary structure. We accomplish this in three steps: (i) extract base pairs from the lattice conformation, (ii) group the extracted base pairs into helices and extend each helix until maximal, (iii) select a disjoint set of base pairs from the maximal helices.

To extract base pairs from the lattice conformation, we use a simple criterion: Two bases are *complementary *if they are either a Watson-Crick pair CG or AU, or a wobble pair GU. Two complementary bases form a *base pair *if they are adjacent in the lattice conformation, and are separated by at least three other bases in the RNA sequence (the hairpin constraint). If two stacking base pairs (*i, j*) and (*i *+ 1, *j *- 1) are both Watson-Crick pairs, then both base pairs are extracted. If a base pair (*i, j*) is a wobble pair and is stacked with two other base pairs (*i *- 1, *j *+ 1) and (*i *+ 1, *j *- 1) that are both Watson-Crick pairs, then all three base pairs are extracted.

Given the initial set of base pairs extracted from the lattice conformation, we group consecutive base pairs into helices. We do not allow bulges or internal loops in the helices. Then each helix can be specified by three numbers (*i*, *j*, ℓ), where (*i, j*) the outer-most base pair in the helix, and ℓ is the helix length, i.e., the number of base pairs in the helix. For example, the helix (4, 12, 3) consists of three stacking base pairs (4, 12), (5, 11), and (6, 10). We then extend each helix into a maximal helix: first extend (*i*, *j*, ℓ) inward to (*i, j*, ℓ + *k*) incrementally, for *k *= 1, 2,..., while the inner-most base pair satisfies the hairpin constraint (*j *- ℓ - *k *+ 1) - (*i *+ ℓ + *k *- 1) > 3 and the helix energy becomes lower; next extend the helix outward, from (*i*, *j*, ℓ') to (*i *- *k*, *j *+ *k*, ℓ' + *k*), in a similar manner. Here the energy of a helix of length ℓ is the sum of the ℓ - 1 (negative) stacking energies of consecutive base pairs.

Recall that each base may be adjacent to two or more complementary bases on the 3D triangular lattice since each lattice point has 12 neighbors. Thus the initial set of base pairs extracted from the lattice conformation may not be disjoint. It follows that the maximal helices obtained from these base pairs may not be disjoint either. We use a greedy algorithm to select a disjoint set of base pairs from these maximal helices. The greedy algorithm is based on a classical concept in graph theory called the page number [53]. Define the *page number *of a secondary structure as the smallest positive integer *k *such that the base pairs in the structure can be partitioned into *k *pseudoknot-free sub-structures. Then a pseudoknot-free secondary structure has page number one, and a simple H-pseudoknot has page number two. RNA secondary structures with page number two have been studied previously as the bi-secondary structures [54,55]. Recent research on the classification of RNA pseudoknots [56,57] has shown that most known RNA secondary structures have page number two, and that almost all known RNA secondary structures have page number at most three. Considering these facts, our greedy algorithm repeats the following selection round until every helix is either assigned to a page or deleted:

1. Among the helices not yet assigned to any page, find one helix *H *with the lowest energy.

2. If *H *does not cross any helices on page 1, assign *H *to page 1; else if *H *does not cross any helices on page 2, assign *H *to page 2; else delete *H*.

3. If *H *is assigned to either page 1 or page 2, trim each unassigned helix to remove its base pairs that overlap with *H*. Delete any unassigned helix that contains less than three base pairs after the trimming.

After the greedy algorithm, the base pairs assigned to either page 1 or page 2 are then output as the predicted secondary structure.

### Reconstructing Tertiary Structure from Predicted Secondary Structure

Our method for tertiary structure reconstruction is based on the same folding simulation approach that we use for secondary structure prediction. In fact all aspects of the annealing procedure remain the same except the scoring function. The new scoring function for reconstruction has two components: the match bonus and the sharp turn penalty. Given a set of base pairs as input, the match bonus includes a constant positive score for each base pair in the input that is realized by two adjacent bases in the lattice conformation. For aesthetic purpose, the sharp turn penalty includes some negative score for each base *i *at which the turn angle of the three consecutive bases (*i *- 1, *i*, *i *+ 1) is either 60° or 90° (120° and 180° are the two other possible angles). Since finding a conformation that realizes all base pairs in the input is more important than minimizing the number of sharp turns in the conformation, we choose the magnitude of penalty for each sharp turn to be a constant (4 for 60° and 1 for 90°) times 1/*L *of the match bonus for a single base pair, where *L *is the length of the RNA sequence. This sharp turn penalty, although very low in magnitude, is still effective in discriminating alternative structures. We observed that the tertiary structures reconstructed by the folding simulation indeed became noticeably smoother with the sharp turn penalty in the scoring function.

### Visualizing Lattice Conformation of RNA Secondary Structure

We implemented a 3D visualization program that, given a sequence of RNA bases and a turn sequence of lattice directions, decodes a lattice conformation of the RNA sequence from the turn sequence, and displays the RNA structure in the *ball-and-stick *model: each base is represented as a ball; two consecutive bases in the sequences are connected by a stick. Optionally, two complementary bases adjacent in the lattice conformation (thus forming a base pair) are connected by a line segment. All figures of RNA structures included in this paper are actual screenshots of our visualization program.

As we would expect from a typical 3D visualization program, the user is able to rotate, zoom, and adjust the 3D view using mouse and keyboard. Moreover, the user can manipulate the RNA structure interactively, by executing pull moves, and save the modified structure in a file. The most attractive feature of our visualization program is perhaps its ability to play RNA folding movies [58,59]. Each frame of animation corresponds to an incremental change to the lattice conformation due to a single pull move. The whole sequence of pull moves for the movie can be read either from a file, in which each line contains two numbers for the base index and the lattice direction of a pull move, or directly from the output of a running annealing procedure, in real time. As we observed in bioinformatics classrooms, the visualization program is a great pedagogic device.

## Software

We implemented a suite of computer programs for RNA folding on the 3D triangular lattice. Our software package consists of the following components:

1. The Delta library delta.c and delta.h, which includes basic data structures and functions for the 3D triangular lattice, pull moves, file input/output, and RNA-specific information such as stacking pair energies. (The name "delta" stands for "a triangular tract of sediment deposited at the mouth of a river" as in the 3D triangular lattice, and for "a finite increment of a variable" as in the score difference of an annealing step.)

2. The folding simulation program fold.c for the prediction of RNA secondary structures and the reconstruction of RNA tertiary structures.

3. The 3D visualization program show.c for displaying the lattice conformations of RNA secondary structures and for playing RNA folding movies.

4. The selection program is.c for selecting disjoint base pairs from the output of folding simulation.

5. Data conversion tools such as db2bp.c (dot brackets to base pairs), bp2hx.c (base pairs to helices), hx2bp.c (helices to base pairs), etc.

6. Traditional UNIX Makefiles for automating program compilation and experiments, and helper programs such as ssa.c (sensitivity, selectivity, and accuracy) for the analysis of experimental results.

**Figure 11:**
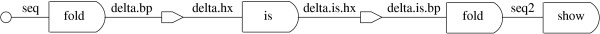
**A typical pipeline of simulation, selection, reconstruction, and visualization**.

We refer to Figure 11 for a typical use of the programs in our software package. Given an RNA sequence, the folding simulation program fold (in prediction mode) finds a good lattice conformation and extract the base pairs, the conversion tool bp2hx converts the base pairs to helices, the selection program is selects a disjoint set of helices, the conversion tool hx2bp converts the helices back to base pairs, the folding simulation program fold (in reconstruction mode) reconstructs a lattice conformation of the RNA sequence as a turn sequence of lattice directions, and finally the 3D visualization program show displays the reconstructed lattice conformation of the predicted structure.

**Figure 12:**
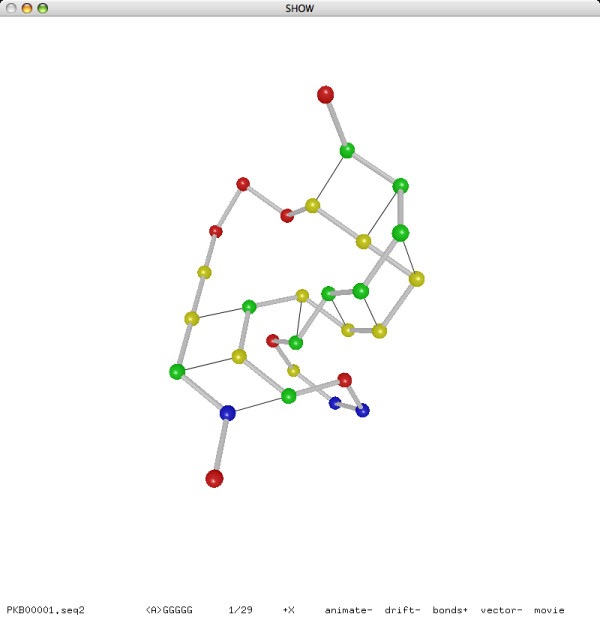
**Example output for **PKB00001 **(29 bases, 9 base pairs)**.

The whole pipeline is encapsulated by the Makefiles in two simple commands. Given an RNA sequence in the file PKB00001.seq, the command make PKB00001.result automatically goes through the pipeline and saves the output to the three files PKB00001.delta.hx, PKB00001.delta.is.hx, and PKB00001.seq2. Then the command make PKB00001.show prints a textual representation of the predicted secondary structures PKB00001.delta.hx (before selection) and PKB00001.delta.is.hx (after selection) to the console, and displays the reconstructed lattice conformation PKB00001.seq2 using the visualization program. We refer to Figure 12 for an example output of the visualization problem. An example output to the console is the following:

AGGGGGGACUUAGCGCCCCCCAAACCGUA

 ______        ______

       __                 __

         __          __

         __           __

             __          __

>

 ______        ______

            ___           ___

To watch the folding simulation of the sequence PKB00001.seq in real time as a movie, run the command make PKB00001.movie.

## Results

To evaluate our RNA folding programs, we use the RNA sequences with known secondary structures from PseudoBase [14]. As of May 25, 2008, the PseudoBase contained 279 partial RNA sequences, of which 252 are consecutive sequences without gaps. We collected these 252 sequences and their known secondary structures (in dot-bracket format) in a FASTA file pseudobase.fasta as the data set for our experiments. The sequences in the data set have maximum length 131 and minimum length 20; the average length is about 43 with a standard deviation of 24.

Three low-end desktop computers were used in our experiments: for pseudoknot prediction, two Dell OptiPlex GX620 with 2.8-3.0 GHz Pentium D Processor and 2 GB RAM running Microsoft Windows XP Professional Service Pack 3 and Cygwin 1.5.25; for reconstruction and visualization, an Apple iMac with 2 GHz PowerPC G5 Processor and 2 GB RAM running Mac OS X 10.4.11.

We compare our RNA pseudoknot prediction method, code named DeltaIS, with the HotKnot algorithm of Ren, Rastegari, Condon and Hoos [15]. HotKnot is an effective heuristics that has been shown [15] to outperform the well-known Pseudoknots algorithm of Rivas and Eddy [60], the NUPACK algorithm of Dirks and Pierce [61], and the Iterated Loop Matching algorithm of Ruan, Stormo and Zhang [62], and to give better results in many cases than the genetic algorithm from the STAR package of van Batenburg, Gultyaev, and Pleij [63] and the pknotsRG-mfe algorithm of Reeder and Giegerich [64].

**Figure 13:**
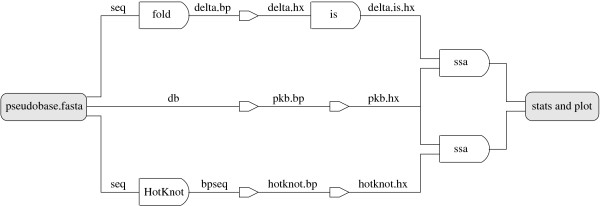
**A flow chart for the pseudoknot prediction experiment**.

We refer to Figure 13 for a flow chart of our experiment on pseudoknot prediction. For each sequence in the data set, the top branch (DeltaIS) computes a secondary structure following the simulation and selection steps of our pipeline in Figure 11. The middle branch (PseudoBase) extracts the known secondary structure of each sequence, and converts it from dot-brackets to base pairs and then to helices. The bottom branch (HotKnot) also computes a secondary structure for each sequence, using HotKnot, and converts it from the bpseq format to our helix format. The secondary structures predicted by DeltaIS and HotKnot are then compared with the known secondary structures from PseudoBase using the analysis program ssa. Since DeltaIS is randomized, we computed 66 different runs of the DeltaIS branch for the analysis, where each run took 20-30 hours to complete. A single run of the (deterministic) HotKnot branch, on the other hand, takes only a few minutes to complete. Thus DeltaIS is not advantageous over HotKnot in terms of speed. Note however that the running time of DeltaIS depends on the tunable parameters of the program. By default, the initial number of annealing steps in each iteration is set to 100·ℓ^2 ^for a sequence of length ℓ. With multiple rounds of doubling steps and with five iterations in each round, the runnning time of DeltaIS on individual sequences ranges from less than a minute (for the shortest sequences of 20 bases) to more than an hour (for some longer sequences of more than 100 bases). This running time can of course be reduced by using fewer annealing steps, but when the number the annealing steps is reduced to a certain point, the prediction accuracy will also be affected.

To compare a predicted secondary structure with a known secondary structure, we count the *true positives *TP as the base pairs that are known and are predicted, the *false negatives *FN as the base pairs that are known but are not predicted, and the *false positives *FP as the base pairs that are not known but are predicted. Then we define three quality measures:

Both sensitivity and selectivity are standard quality measures that are widely used. We introduce the measure of accuracy here as a combination of sensitivity and selectivity. Intuitively, consider the predicted secondary structure and the known secondary structure as two sets of base pairs, then the measure of accuracy is the intersection of the two sets over the union of the two sets.

We first compare the overall performances of DeltaIS and HotKnot. For this we calculate the three quality measures (sensitivity, selectivity, and accuracy) using the equations above with the three numbers (true positives, false negatives, and false positives) summed over all 252 sequences in the data set. Since Delta is randomized, we first compute the three measures using the best prediction of 66 runs for each sequence, then compute the averages and standard deviations of the measures over the 66 runs. The results are shown in Table 2. From the results we can see that DeltaIS is comparable to HotKnot in selectivity and outperforms HotKnot in both sensitivity and accuracy. Also note that the best and the average are very close for DeltaIS. This shows that the performance of DeltaIS is consistent over different runs.

**Table 2:** DeltaIS versus HotKnot on sensitivity, selectivity, and accuracy.

	Sensitivity	Selectivity	Accuracy
DeltaIS (best of 66 runs)	80.20%	78.37%	65.67%
DeltaIS (average ± stdev)	79.11 ± 0.82%	77.39 ± 0.83%	64.26 ± 1.09%
HotKnot	71.69%	78.47%	59.90%

**Figure 14:**
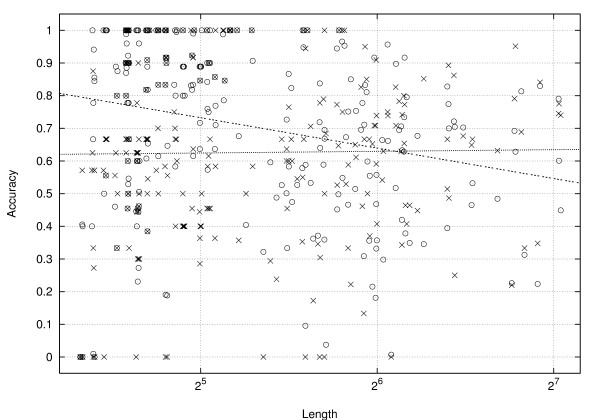
**A scatter plot of the prediction accuracies of DeltaIS (⊙) and HotKnot (×) on individual sequences**.

We next compare the performances of DeltaIS and HotKnot on individual sequences. Refer to Figure 14 for a scatter plot in which each of the 252 sequences corresponds to a point ⊙ for DeltaIS and a point × for HotKnot, with the sequence length (in log scale) as the *x*-coordinate and with the prediction accuracy (DeltaIS accuracy averaged over the 66 runs) as the *y*-coordinate. There are no clear patterns on the distribution of these data points, but linear regressions of the two point sets produce an almost horizontal line for HotKnot and a slightly decreasing line for DeltaIS. The intersection point of the two lines corresponds to the sequence length of about 70. This implies that the improvement of DeltaIS over HotKnot in overall prediction accuracy is especially pronounced on the shorter sequences.

After analyzing the scatter plot of prediction accuracies for individual sequences, we count the number of sequences whose secondary structures were predicted perfectly, with all three measures sensitivity, selectivity, and accuracy equal to 100%. Out of the 252 sequences in the data set, HotKnot predicted the secondary structures of 36 sequences perfectly. In contrast, DeltaIS predicted the secondary structures of 47 sequences perfectly in all 66 runs (that is, the secondary structure of each of the 47 sequences was predicted perfectly in each of the 66 runs). Moreover, DeltaIS predicted the secondary structures of 82 sequences perfectly in at least one of the 66 runs (note that these 82 sequences account for almost one third of the 252 sequences in the data set). Among the 36 sequences whose secondary structures are predicted perfectly by HotKnot, 21 sequences are included in the 47 sequences whose secondary structures are predicted perfectly by HotKnot in all 66 runs, and 30 sequences are included in the 82 sequences whose secondary structures are predicted perfectly by HotKnot in at least one of the 66 runs. The overlaps do not seem big enough to signify any strong correlation between the prediction accuracies of DeltaIS and HotKnot on individual sequences.

**Figure 15:**
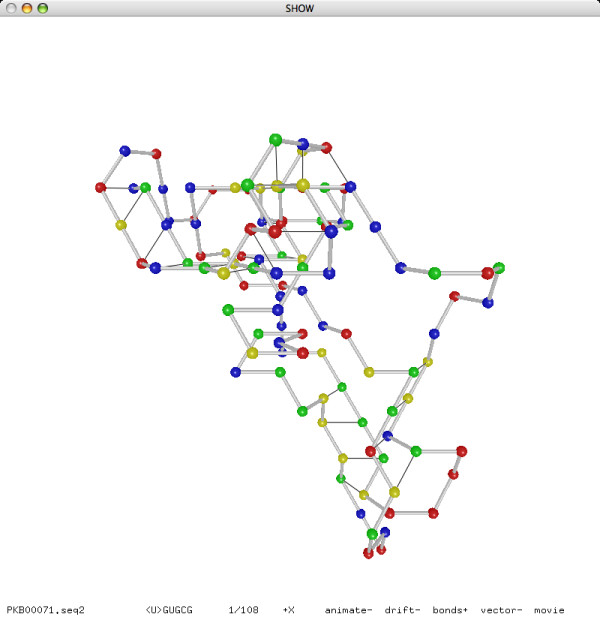
**Reconstructed lattice conformation of **PKB00071 **(108 bases, 24 base pairs)**.

**Figure 16:**
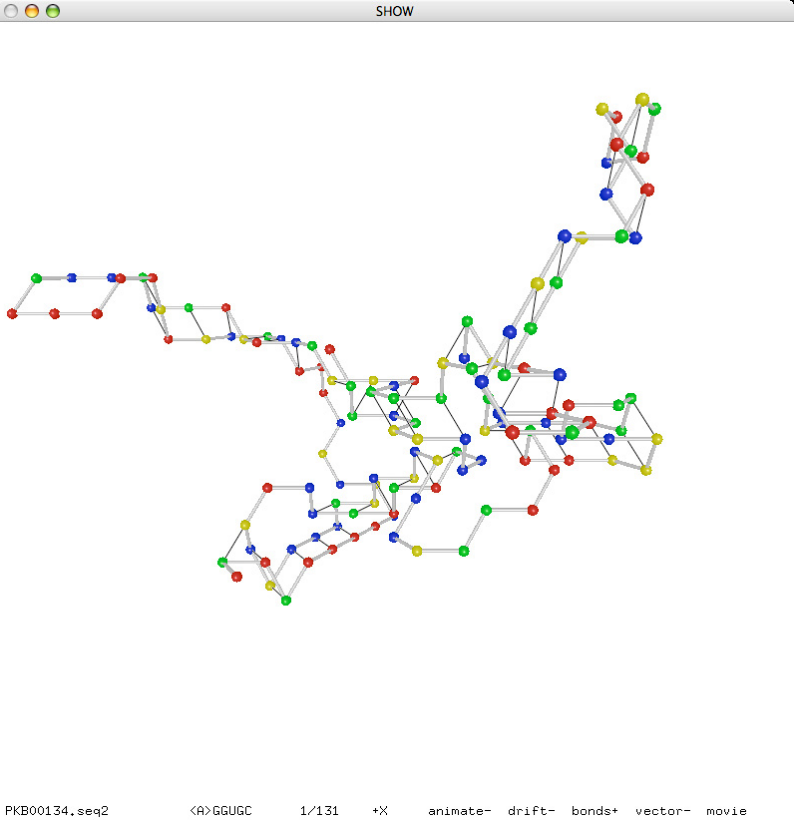
**Reconstructed lattice conformation of **PKB00134 **(133 bases, 44 base pairs)**.

In addition to the experiment on pseudoknot prediction, we also performed an experiment on reconstruction. Our reconstruction method, based on the same folding simulation approach as our prediction method, can successfully reconstruct the lattice conformations of all 252 sequences from their known secondary structures in less than 15 minutes. Moreover, most of the sequences need only one iteration of the annealing procedure for a successful reconstruction. We refer to Figures 15 and 16 for the reconstructed lattice conformations of two sequences.

## Discussion

### Reconstruction Versus Prediction

Our methods for RNA tertiary structure reconstruction and RNA secondary structure prediction are based on the same folding simulation approach. The experimental results, however, show that reconstruction is much easier than prediction: for reconstruction, 100% success rate in less than 15 minutes; for prediction, 79.11% sensitivity 77.39% selectivity 64.26% accuracy in 20-30 hours. We give two explanations for this disparity:

1. The 3D triangular lattice is a dense regular lattice that approximates the 3D space very well. By a comparison of the sequence space and the shape space [11], one can show that for any given RNA sequence, the number of all possible conformations on the 3D triangular lattice far exceeds the number of all possible secondary structures as sets of disjoint base pairs. Thus each distinct secondary structure has many corresponding lattice conformations. The abundance of "witnesses" for each secondary structure makes it easy for a random search to find a corresponding lattice conformation.

2. The annealing procedure is a biased search. As remarked by Zwanzig, Szabo and Bagchi [65] on Levinthal's paradox, it is difficult for an uneducated monkey to type a verse of Shakespeare accidentally, but if the letters typed correctly are forever locked in place while incorrect letters can still be changed, then the chance of such a feat becomes significantly higher. The same logic explains why our reconstruction method based on folding simulation is so effective. Indeed a similar argument also explains the effectiveness of Hofacker et al.'s heuristic for the RNA design problem [23].

An interesting aspect of our approach is that it can be used to complement the existing methods. Given a secondary structure predicted by any other method, we can first reconstruct a lattice conformation of the structure, then obtain an improved structure by folding simulation using the old structure as the starting point.

### Multiple Predictions

The random nature of our folding simulation approach may cause different iterations of the annealing procedure to find different best structures, although our experiment showed that the predictions are typically consistent (recall that DeltaIS predicted the secondary structures of 47 sequences perfectly in each of 66 different runs). This may seem like a disadvantage, if our goal is simply to predict a unique structure. The biological reality, however, is not so simple. The disadvantage may turn out to be a desirable feature in certain scenarios. Some RNA sequences are "switches" in the sense that they can assume several very different meta-stable structures. The conformation spaces and folding trajectories of such RNA sequences cannot be studied adequately with the thermodynamic approach alone, which aims at a supposedly unique best structure with the minimum free energy. Flamm, Fontana, Hofacker and Schuster [12] showed an effective kinetic approach based on elementary-step simulation. One of their examples for illustrating the kinetic approach is an RNA switch with two highly stable secondary structures:

GGCCCCUUUGGGGGCCAGACCCCUAAAGGGGUC

((((((((((((((:::::))))))))))))))

((((((::::)))))):((((((::::))))))

**Figure 17:**
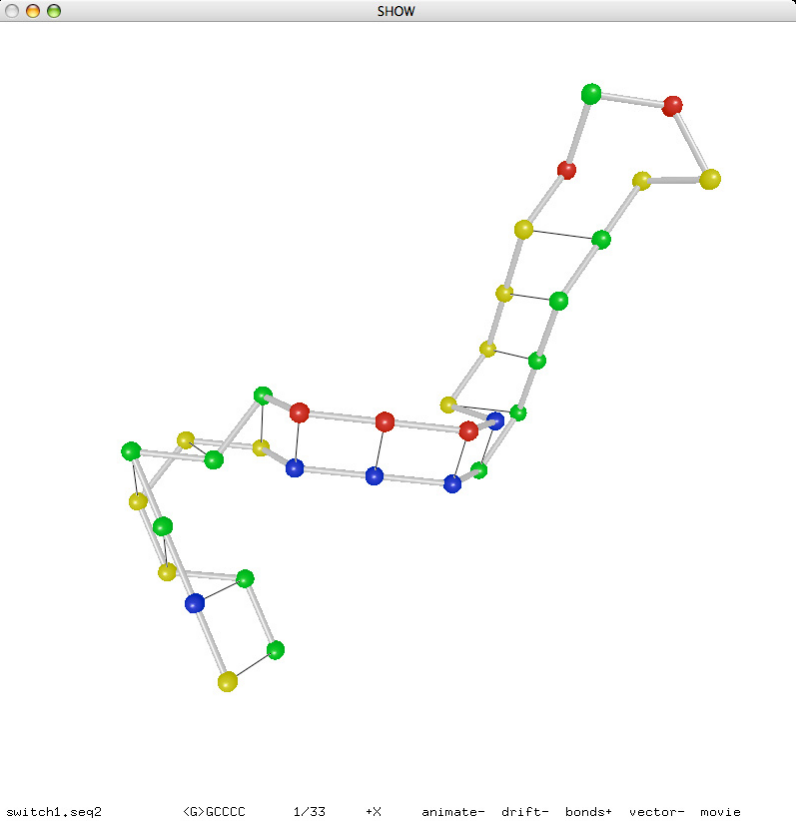
**The 1st secondary structure of an RNA switch**.

**Figure 18:**
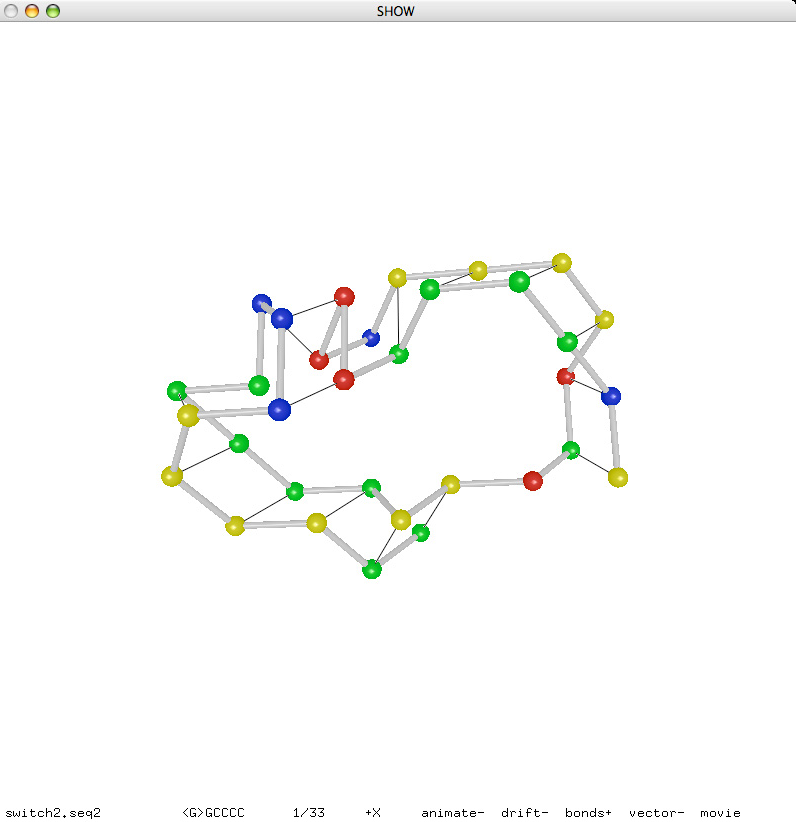
**The 2nd secondary structure of an RNA switch**.

Using our folding simulation program on this sequence, we were able to predict both structures within five iterations of the annealing procedure. The lattice conformations are shown in Figures 17 and 18. Note that the first structure is a stem-loop and the second structure is indeed a kissing-hairpin:

GGCCCCUUUGGGGGCCAGACCCCUAAAGGGGUC

((((((((((((((:::::))))))))))))))

(((((([[[:)))))):((((((:]]]))))))

Although the two structures are very different, they have roughly the same number of base pair stackings and hence very close scores in the folding simulation. We recommend that the users of our program always run the prediction program multiple times on each sequence and consider each predicted structure as a possible candidate.

### Longer Sequences

As we observed from the scatter plot in Figure 14, the prediction accuracy of DeltaIS tends to deteriorate as the sequence length increases. We believe the reason is the following. Each pull move relocates on average only a constant number of bases. While this semi-local property is desirable for making individual annealing steps efficient, it also means that the average impact of a single pull move on the lattice conformation becomes less significant for longer sequences. As a result, the annealing procedure becomes less capable of escaping local optima. Moreover, longer sequences typically have a much larger number of local optima than shorter sequences. Possible ways to improve the prediction accuracy for long sequences are (i) to design a more effective move than the pull move, (ii) to combine several pull moves into an atomic unit as a combo move, and (iii) to use alternative Monte Carlo schemes such as simulated tempering [66] instead of simulated annealing. We will try these ideas in our future work.

## Conclusion

Folding simulation on the 3D triangular lattice is effective method for RNA secondary structure prediction and tertiary structure reconstruction. The visualization software for the lattice conformations of RNA structures is a valuable tool for the study of RNA folding and is a great pedagogic device.

## Availability

Our RNA folding programs have been tested on three major platforms: Microsoft Windows (Cygwin), Linux, and Mac OS X. The complete source code, documentation, data set, and experimental results can be downloaded from the companion website of this paper: RNA folding on the 3D triangular lattice http://www.cs.usu.edu/~mjiang/rna/DeltaIS/.

## Authors' contributions

MJ conceived of the study and designed the algorithms, the software, and the experiments. MM and MJ implemented a basic version of the software and performed initial experiments in a preliminary study. JG and MJ improved the accuracy of the RNA secondary structure prediction method, optimized the source code, revised the documentation, and created the companion website. All authors read and approved the final manuscript.
